# Annual Meeting of the Henry County Medical Society

**Published:** 1856-07

**Authors:** A. A. Dunn, T. D. Fitch

**Affiliations:** President; Secretary


*Annual Meeting of the Henry County Medical Society, held at Kewanee, June 5th*, 1856.

Dr. E. Pomeroy, President, being absent, Dr. J. M. Winn, Vice-President, was called to the chair.

The minutes of the previous meeting were read and approved.

The following physicians, being present, were proposed as proper persons to become members of this Society.

C. H. Gran, M.D., of Andover, proposed by Dr. O. H. Edwards.

A. A. Dunn, M.D., Cambridge, by Dr. H. Ludington.
M. G. Lyman, M.D., Geneseo, ditto.
J. R. Hays, M.D., Rock Island, ditto.
H. C. Morey, M.D., Geneseo, ditto.
G. P. Dimmitt, M.D., Cambridge, ditto.
N. Holton, M.D., Buda, by Dr. Fitch.
E. Gaylord, M.D., Kewanee, ditto.
J. S. Lamper, M.D., Bradford, by Dr. H. Ludington.

Applications were referred to Censors, who reported favorably.

On motion of Dr. Fitch they were received as members.

Society proceeded to the election of officers for the ensuing year, which resulted as follows:—

For President, A. A. Dunn, of Cambridge.
For Vice-President, R. J. Stough, Geneseo.
For Secretary and Treasurer, T. D. Fitch, Kewanee.
M. G. Lyman, Geneseo, C. H. Gran, Andover, J. M. Winn, Wethersfield, Censors.

The Annual Address by Dr. Ludington, on the Rise and Progress of the Science of Medicine.

On motion of Dr. Hays, of Rock Island, a vote of thanks was paid Dr. Ludington for his address.

Dr. G. M. Austin, of Kewanee, was proposed by Dr. Fitch; whose proposition was referred to the Censors, who reported favorably. On motion of Dr. Fitch, Dr. Austin was admitted a member.

The Committee on Fee Bill reported, and, on motion of Dr. Winn, it was taken up *seriatim*, which resulted as follows:—

Town visits, $1.
Each additional visit same day, 50c.
Office prescriptions, with or without medicine, 50c. to $5.
Country visits, first mile, $1, each additional mile, 50c.
Night visits, in addition to the above, 50 per cent.
Consultation visits in town, $5; in country, mileage.
Obstetrical cases in town, from $5 to $10.
In the country, mileage additional.

Operations in minor surgery, from 50c. to $5.
Reducing dislocations, from $3 to $15.
Amputations, from $5 to $200.
Venereal cases, from $5 to $50.
Capital operations, from $25 to $500.

MEMBERS AND SUBSCRIBERS.

| | | |
|---|---|---|
| A. A. Dunn, | M. G. Lyman, | H. C. Morey, |
| R. J. Stough, | C. H. Gran, | J. R. Hays, |
| T. D. Fitch, | J. M. Winn, | G. P. Dimmitt, |
| N. Holton, | G. M. Austin, | E. Gaylord, |
| H. Ludington, | J. G. Lamper, | O. H. Edwards. |

Report of cases by Drs. Dunn, Stough, Fitch, Gran, and Hays.

The following resolution by Dr. Ludington, was read and adopted *una voce*:—

1st. That 500 pamphlets be printed, containing the Constitution and By-Laws of the Henry County Medical Society; the minutes of this meeting; the tariff of charges, with each member's name attached, and the Code of Ethics. The expense to be mutually borne, and each member furnished his proportion of said pamphlets.

2d. That the minutes of this meeting, and the tariff of charges with the names attached, be published in the county papers, and the *N.-W. Medical and Surgical Journal*.

On motion of Dr. Stough, Society adjourned to meet in Geneseo, on the first Monday in September, 1856, at 10 o'clock, A.M. A. A. DUNN, M.D., *President*.

T. D. FITCH, M.D., *Secretary*.

